# Growth and remodeling of the dissected membrane in an idealized dissected aorta model

**DOI:** 10.1007/s10237-023-01782-7

**Published:** 2023-11-10

**Authors:** Lise Gheysen, Lauranne Maes, Nele Famaey, Patrick Segers

**Affiliations:** 1 Institute for Biomedical Engineering and Technology, Electronics and Information Systems, Ghent University, Ghent, Belgium; 2 Biomechanics Section, Mechanical Engineering, KU Leuven, Leuven, Belgium

**Keywords:** Aortic dissection, Finite element analysis, Growth and remodeling, Membrane thickening, Parameter space reduction

## Abstract

While transitioning from the acute to chronic phase, the wall of a dissected aorta often expands in diameter and adaptations in thickness and microstructure take place in the dissected membrane. Including the mechanisms, leading to these changes, in a computational model is expected to improve the accuracy of predictions of the long-term complications and optimal treatment timing of dissection patients. An idealized dissected wall was modeled to represent the elastin and collagen production and/or degradation imposed by stress- and inflammation-mediated growth and remodeling, using the homogenized constrained mixture theory. As no optimal growth and remodeling parameters have been defined for aortic dissections, a Latin hypercube sampling with 1000 parameter combinations was assessed for four inflammation patterns, with a varying spatial extent (full/local) and temporal evolution (permanent/transient). The dissected membrane thickening and microstructure was considered together with the diameter expansion over a period of 90 days. The highest success rate was found for the transient inflammation patterns, with about 15% of the samples leading to converged solutions after 90 days. Clinically observed thickening rates were found for 2–4% of the transient inflammation samples, which represented median total diameter expansion rates of about 5 mm/year. The dissected membrane microstructure showed an elastin decrease and, in most cases, a collagen increase. In conclusion, the model with the transient inflammation pattern allowed the reproduction of clinically observed dissected membrane thickening rates, diameter expansion rates and adaptations in microstructure, thus providing guidance in reducing the parameter space in growth and remodeling models of aortic dissections.

**Supplementary Information:**

The online version contains supplementary material available at 10.1007/s10237-023-01782-7.

## Introduction

Similar to the healthy aorta, the dissected aortic wall evolves over time as a consequence of soft tissue growth and remodeling. This typically leads to aortic expansion as well as thickening and a reduced motion of the dissected membrane, i.e. the delaminated part of the aortic wall that separates the true lumen (normal blood path) from the false lumen (newly formed blood path) between the delaminated and the remaining part of the wall (Karmonik et al. 2012; Peterss et al. 2016; Trimarchi et al. 2013). Moreover, inflammation has been observed in the region of the dissection, which is expected to affect the soft tissue growth and remodeling (He et al. 2006; Luo et al. 2009; Wang et al. 2020). Understanding the impact of growth and remodeling in the context of aortic dissections might contribute to treatment optimization. Indeed, both medically and endovascularly treated patients regularly show the development of complications, such as aortic expansion and malperfusion, and/or the need for late (re)interventions (Fattori et al. 2013; Nienaber et al. 2011). This indicates that the current treatment strategies are often not able to determine the optimal patient-specific treatment at hospital admission. Moreover, the timing of the intervention has been found to impact the long-term outcome of the treatment in terms of maximal aortic diameter, false lumen size and true lumen expansion (Jubouri et al. 2022). In combination with accurate models of the blood flow and thrombus formation, the inclusion of growth and remodeling in models of aortic dissection will help in the prediction of potential complications and/or the preferred timing of stent-graft placement.

Multiple theoretical frameworks to represent soft tissue growth and remodeling, such as the (homogenized) constrained mixture theory, have been proposed over the past decades and applied to arterial diseases as hypertension and aneurysm formation, where the growth and remodeling process was explained as a consequence of elastin degradation and collagen remodeling induced by changes in stress and/or inflammation (Braeu et al. 2017; Brandstaeter et al. 2021; Cyron et al. 2014; Horvat et al. 2019; Latorre et al. 2019; Maes et al. 2023; Mousavi et al. 2019; Ramachandra et al. 2020). Furthermore, a multi-scale framework was developed and used to model the effect of the microstructural constituent remodeling on the macroscopic soft tissue deformation in a chronic dissection (Gacek et al. 2023).

However, these growth and remodeling models come with a large set of parameters and knowledge regarding proper physiological values thereof is still limited. In case longitudinal experimental data are available, values for the considered parameters can be determined through parameter fitting (Maes et al. 2023). However, often insufficient or even no data are available and parameter values have to be assumed. In this respect, the impact of adopted parameter values for the stress-induced growth and remodeling has been tested, but not in the framework of aortic dissections (Braeu et al. 2017; Brandstaeter et al. 2021; Cyron et al. 2014; Horvat et al. 2019; Mousavi et al. 2019; Valentin and Humphrey 2009). Indeed, the transition from the acute to the chronic phase in the dissected aorta has, to the authors’ knowledge, not yet been modeled and proper parameter values have, consequently, not yet been established.

Therefore, this study aims to investigate whether the transition of the dissected aorta from the acute to the chronic phase, and the corresponding dissected membrane thickening, can be represented by applying stress- and inflammation-mediated growth and remodeling of elastin and collagen using reported ranges for the growth and remodeling parameters.

## Methods

### Model framework

#### Geometry

**Fig. 1:**
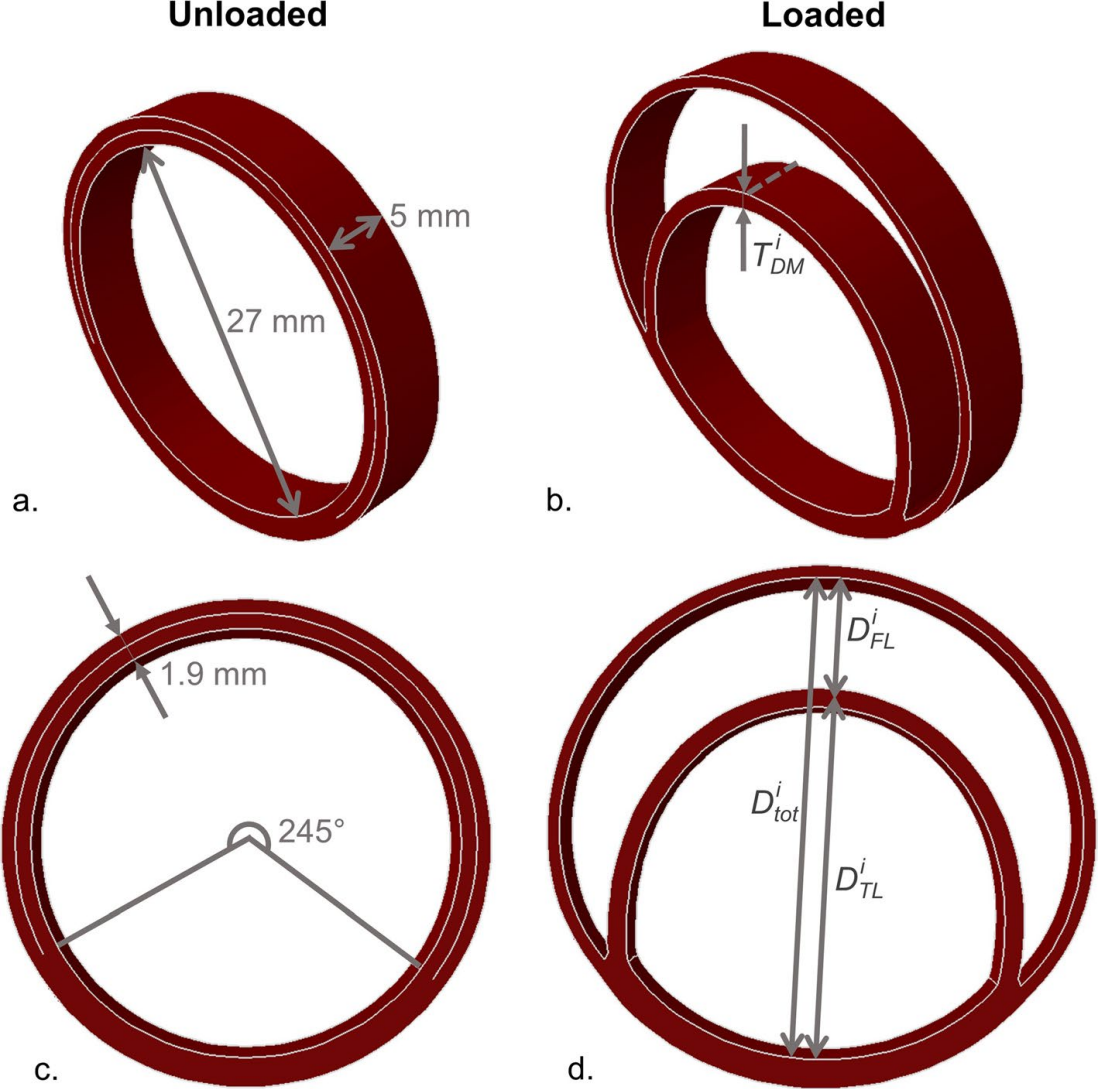
Overview of the slice model in the (**a**, **c**) unloaded and (**b**, **d**) loaded configuration. The predefined dimensions of the model are indicated on the (**a**, **c**) unloaded shape, which corresponds to the geometry of the in vivo healthy wall, but already includes the predefined location of the false lumen. A schematic indication of the measured geometrical parameters is illustrated on the (**b**, **d**) loaded configuration, with ${T}_{DM}^{i}$ being the dissected membrane thickness and ${D}_{tot}^{i}$, ${\mathrm{\it{D}}}_{\mathrm{\it{FL}}}^{\mathrm{\it{i}}}$ and ${D}_{TL}^{i}$ referring to the respective total, false lumen and true lumen diameter, where day *i* indicates the day of interest in the growth and remodeling process. Note that ${D}_{tot}^{i}$, ${D}_{FL}^{i}$ and ${D}_{TL}^{i}$ are measured at the same line, but are indicated next to each other for better visualization

The reference geometry is based on an idealized model of a slice of a dissected aorta. The dissected wall model was developed as a cylindrical geometry with an axial length of 5 mm, an inner diameter of 27.3 mm and a total wall thickness of 1.90 mm (Fig. 1) (Yamauchi et al. 2018). Of the total wall thickness, a fraction of 75% was assumed to correspond to the media and 25% to the adventitia (Humphrey 2013; Weisbecker et al. 2012). The false lumen is implemented in the cylindrical reference geometry by integrating unconnected elements at 70% of the medial layer, thus in the outer third of the media, and comprises 245° of the circumference, a value retrieved from a patient CT scan and in correspondence to Brunet et al. (2023) (Osada et al. 2013). The CT scan was acquired from the University Hospital of Düsseldorf with consent of the local ethical committee (reference number: 2017064325) (Logghe et al. 2021). In its unloaded state, the false lumen is not visible and the model appears as the geometry of a healthy cylindrical aortic wall (Fig. 1(a, c)). The model does not include the tears or connection with the healthy aortic wall and, therefore, represents an infinitely long dissection.

#### Acute material behavior

Both the medial and the adventitial layer of the dissected wall model are assumed to behave as an anisotropic hyperelastic Holzapfel-Gasser-Ogden (HGO) material with two non-dispersed fiber families (Holzapfel et al. 2000). The strain energy density functions for the elastin and collagen fraction of each layer, ${\psi }_{M}^{e}$, ${\psi }_{M}^{c}$ and ${\psi }_{A}^{e}$ and ${\psi }_{A}^{c}$, were, consequently, defined as

$${\psi }_{M}^{e}=\frac{{c}_{10M}}{{\rho }_{0M}^{e}}\left({I}_{1}-3\right); {\psi }_{M}^{c}=\frac{{k}_{1M}}{{\rho }_{0M}^{c}}\frac{1}{{2k}_{2}}\left(\sum_{i=\mathrm{4,6}}{\mathrm{e}}^{{{k}_{2}\left({I}_{iM}-1\right)}^{2}}-1\right)$$

$${\psi }_{A}^{e}=\frac{{c}_{10A}}{{\rho }_{0A}^{e}}\left({I}_{1}-3\right); {\psi }_{A}^{c}= \frac{{k}_{1A}}{{\rho }_{0A}^{c}}\frac{1}{{2k}_{2}}\left(\sum_{i=\mathrm{4,6}}{\mathrm{e}}^{{{k}_{2}\left({I}_{iA}-1\right)}^{2}}-1\right)$$

1 $$\mathrm{with}\text{ }{c}_{10A}=0.34{c}_{10M}\text{ }\mathrm{ and }\text{ }{k}_{1A}=1.17{k}_{1M},$$

where subscripts *M* and *A*, respectively, indicate the medial and adventitial layer and superscripts *e* and *c* refer to elastin and collagen, respectively. Parameters *c*_*10*_, *k*_*1*_ and *k*_*2*_ correspond to the elastin shear modulus, the collagen stiffness and the collagen fiber stiffening, respectively. Parameters *c*_*10*_ and *k*_*1*_ were linked for the media and adventitia based on the observed elastin and collagen area fractions in both layers (Iliopoulos et al. 2009). The invariants of the right Cauchy-Green tensor ***C*** of deformation gradient tensor ***F***, with ***C*** = ***F***^*T*^***F***, are indicated as *I*_*1*_ and *I*_*i*_, with *i* = 4, 6. The fourth and sixth invariants, *I*_*4*_ and *I*_*6*_, depend on the fiber stretch and the mean fiber angles *α*_*M*_ and *α*_*A*_, which are defined with respect to the circumferential direction and differ for the media and adventitia. Note that the fibers are assumed to contribute to the load bearing in tensile stretch only. The initial density of constituent *i*, expressed as fraction w.r.t. the initial configuration, is accounted for by ${\rho }_{0}^{i}$. Including this density allows the expression of the fraction of collagen and elastin with respect to a reference unit area within each material layer, which is assumed to contain no other constituents than collagen and elastin.

The parameters were determined based on a predefined value of the pulse wave velocity as proposed in Gheysen et al. (2023), where the pulse wave velocity of a full factorial design of the Gasser-Ogden-Holzapfel (GOH) parameters, including collagen fiber dispersion, was calculated for a reference cylinder. A pulse wave velocity of 6 m/s was assumed, which is representative for the aortic stiffness in middle-aged humans and, thus, corresponds to the average age of dissection patients (Logghe et al. 2021). As a non-dispersed one-dimensional fiber orientation was assumed in the growth and remodeling implementation (Sect. 2.2.1), the GOH parameter combination that fulfilled the zero dispersion condition, both in the media and adventitia, and was closest to the target pulse wave velocity of 6 m/s was selected. This resulted in parameters *c*_*10M*_ = 0.015 MPa, *k*_*1M*_ = 0.12 MPa, *k*_*2M*_ = 6.9, *α*_*M*_ = 0.79 rad for the media and *c*_*10A*_ = 0.0051 MPa, *k*_*1M*_ = 0.14 MPa, *k*_*2M*_ = 6.9, *α*_*M*_ = 1.4 rad for the adventitia. The aortic wall tissue was assumed to be composed of elastin and collagen; smooth muscle cells were not accounted for. Consequently, the initial fractions of collagen and elastin were determined as percentage of the total elastin and collagen area, based on the data of Iliopoulos et al. (2009). This resulted in a respective initial collagen fraction for the media and adventitia of 0.27 and 0.40, for each fiber family. The corresponding initial elastin fractions were 0.46 for the medial and 0.20 for the adventitial layer.

#### Loading and boundary conditions

To account for the in vivo loading state of the aortic wall, an axial stretch and circumferential and radial deposition stretches have been included using a MATLAB (The MathWorks Inc., USA) implementation of the deposition stretch algorithm of Famaey et al. (2018). This pre-stressing algorithm was first applied to a healthy cylindrical geometry, without false lumen, with a constant deposition strain of 10% in the fiber direction for collagen and in the axial direction for elastin (Bellini et al. 2014; Horny et al. 2014). The in vivo deformation of the acute dissection was subsequently obtained by applying the resulting deposition stretches, obtained from the deposition stretch algorithm in MATLAB, simultaneous with a diastolic pressure of 80 mmHg to the true and false lumen to a finite element model, with 3000 hexahedral elements, in Abaqus/Standard (Dassault Systèmes, France). The healthy configuration was not explicitly determined in the finite element analysis. Note that no load was applied to the dissected membrane, due to the assumption that the pressure exerted by the true and false lumen compensates each other. The slight difference in surface on which the pressure is acting indicates that the applied loading conditions provide an approximation of the in vivo situation. At the proximal and distal boundaries, only radial expansion was allowed for the adventitial layer and the connected medial layer, while the circumferential direction was an additional degree of freedom for the dissected membrane. This procedure leads to a dissected wall, shown in Fig. 1(b, d), that embeds initial stresses in the (remaining) aortic wall, while stress is relieved in the dissected membrane as the delamination takes place and the diastolic pressure is applied.

### Transition from acute to chronic dissection

While the medical consensus on the definition of the acute, subacute and chronic phase of the dissection is, respectively, 0 to 14 days, 2 weeks to 3 months and more than 3 months after onset of the symptoms, a different definition was adopted for the experimental data of Peterss et al. (2016), where the dissected membrane thickening from the acute to the chronic phase was assessed (Dake et al. 2013). Indeed, they considered the membrane thickening after 2 to 6 weeks as subacute and after 6 weeks as chronic. In order to allow for a comparison with the experimental data, the definition of Peterss et al. (2016) was adopted here. In total, 30 growth steps were implemented, each accounting for a time step of 3 days, thus considering growth and remodeling during 90 days.

#### Homogenized constrained mixture theory

The evolution of the dissected wall during the transition from an acute to a chronic dissection was modeled using an implementation of the homogenized constrained mixture algorithm in Abaqus/Standard. While the details of the theory, developed by Cyron et al. (2016), and the implementation, proposed by Maes et al. (2023) and Maes and Famaey (2023), have been extensively described before, the most important aspects are summarized below.

In the homogenized constrained mixture theory, the total deformation gradient tensor ***F*** at time *s* is the result of the soft tissue growth ***F***_***g***_ and the constituent-specific remodeling ${{\varvec{F}}}_{{\varvec{r}}}^{{\varvec{i}}}$ and elastic deformation ${{\varvec{F}}}_{{\varvec{e}}{\varvec{l}}{\varvec{a}}{\varvec{s}}}^{{\varvec{i}}}$ according to

2 $${\varvec{F}}\left(s\right)={{\varvec{F}}}_{{\varvec{e}}{\varvec{l}}{\varvec{a}}{\varvec{s}}}^{{\varvec{i}}}\left(s\right){{\varvec{F}}}_{{\varvec{r}}}^{{\varvec{i}}}{\left(s\right){\varvec{F}}}_{{\varvec{g}}}\left(s\right),$$

with *i* indicating the considered constituent and *i* = *e*, *c* corresponding to elastin and collagen, respectively. Consequently, the elastic deformation gradient tensor ***F***_***elas***_ is determined if the total deformation, which can be determined using a finite element model, and the deformation due to growth and remodeling is known. The soft tissue growth is assumed to occur in the radial direction and to affect the different constituents in the same manner. Therefore, the ***F***_***g***_ at time *s* was implemented as

3 $${\varvec{F}}_{{\varvec{g}}} \left( s \right) = { }\frac{{\rho^{tot} \left( s \right)}}{{\rho^{tot} \left( 0 \right)}}{\varvec{a}}_{{\varvec{g}}} \otimes {\varvec{a}}_{{\varvec{g}}} + \left( {{\varvec{I}} - {\varvec{a}}_{{\varvec{g}}} \otimes {\varvec{a}}_{{\varvec{g}}} } \right),$$

where ${{\varvec{a}}}_{{\varvec{g}}}$ is a unit vector that indicates the main growth direction, ${\rho }^{tot}$ the total density of elastin and collagen together and ${\varvec{I}}$ the identity matrix (Braeu et al. 2017; Maes and Famaey 2023).

As it is assumed that elastin does not remodel, the deformation imposed by the remodeling only depends on the collagen and can be extracted from

4 $${\varvec{F}}_{{\varvec{r}}}^{{\varvec{c}}} \left( {\text{s}} \right) = { }\lambda_{r}^{c,f} \left( s \right){\varvec{M}}^{{\varvec{f}}} \otimes {\varvec{M}}^{{\varvec{f}}} + { }\frac{1}{{\sqrt {\lambda_{r}^{c,f} \left( s \right)} }}\left( {{\mathbf{I}} - {\varvec{M}}^{{\varvec{f}}} \otimes {\varvec{M}}^{{\varvec{f}}} } \right),$$

for one-dimensional collagen fibers (Braeu et al. 2017; Cyron et al. 2016; Maes and Famaey 2023). The mean collagen fiber direction in the initial configuration is indicated by ${{\varvec{M}}}^{{\varvec{f}}},$, while ${\lambda }_{r}^{c,f}(s)$ represents the collagen remodeling stretch in the fiber direction of fiber family *f* at time *s*. For more details about the derivation of this equation, the reader is referred to the original papers (Braeu et al. 2017; Cyron et al. 2016; Maes and Famaey 2023).

The densities of collagen and elastin change over time due to the constituent degradation and, in case of collagen, production. Therefore, the current change in density of constituent *i*, ${\dot{\rho }}^{i}\left(s\right)$, is determined as

5 $${\dot{\rho }}^{i}\left(s\right)={\dot{\rho }}_{+}^{i}\left(s\right)+{\dot{\rho }}_{-}^{i}\left(s\right),$$

where ${\dot{\rho }}_{+}^{i}\left(s\right)$ and ${\dot{\rho }}_{-}^{i}\left(s\right)$ represent the respective production and degradation of constituent *i* at time *s*. Note that the densities, and the corresponding changes, are expressed relative to the initial configuration.

The total strain energy density function ${\psi }^{tot}$ is calculated as a combination of the strain energy density function of the different constituents and their corresponding densities. Moreover, it is assumed that the strain energy only depends on the elastic part of the deformation. This results in

6 $${\psi }_{j}^{tot}\left(s\right)= \sum\limits_{i}{\rho }^{i}\left(s\right){\psi }_{j}^{i}\left({{\varvec{F}}}_{{\varvec{e}}{\varvec{l}}{\varvec{a}}{\varvec{s}}}^{{\varvec{i}}}\left(s\right)\right),$$

where *j* = *M, A* depending on whether the medial or adventitial layer is considered (Sect. 2.1.2).

#### Stress-mediated growth and remodeling

As the aortic wall attempts to restore homeostasis, collagen is remodeled by sensing the difference between the current and the homeostatic stress state (Humphrey and Rajagopal 2002). It is assumed that this stress-mediated growth and remodeling is present, both in health and disease, and, consequently, in the framework of aortic dissections. Therefore, the collagen production depends on the difference between the homeostatic and the current stress in the direction of fiber family *f*, ${\sigma }_{h}^{c,f}$ and ${\sigma }^{c,f}(s)$, respectively, and the gain factor ${k}_{\sigma }^{c}$. The homeostatic fiber stress, ${\sigma }_{h}^{c,f}$, is determined as the stress that corresponds to the imposed homeostatic collagen fiber deposition strain of 10%. The degradation rate is, on the contrary, assumed to be constant and to depend on the characteristic time of collagen *T*^*c*^, which was taken as 101 days (Braeu et al. 2017; Maes et al. 2023). This results in

7 $${\dot{\rho }}_{+}^{c,f}\left(s\right)= \frac{{\rho }^{c,f}(s)}{{T}^{c}} \left(1+ {k}_{\sigma }^{c}\frac{{\sigma }^{c,f}(s)- {\sigma }_{h}^{c,f}}{{\sigma }_{h}^{c,f}}\right)$$

8 $${\dot{\rho }}_{-}^{c,f}\left(s\right)=- \frac{{\rho }^{c,f}\left(s\right)}{{T}^{c}}.$$

This stress-mediated growth and remodeling, thus, retains the homeostatic configuration for a healthy aortic wall, which is indicated in appendix 1.

#### Inflammation-mediated growth and remodeling

**Fig. 2:**
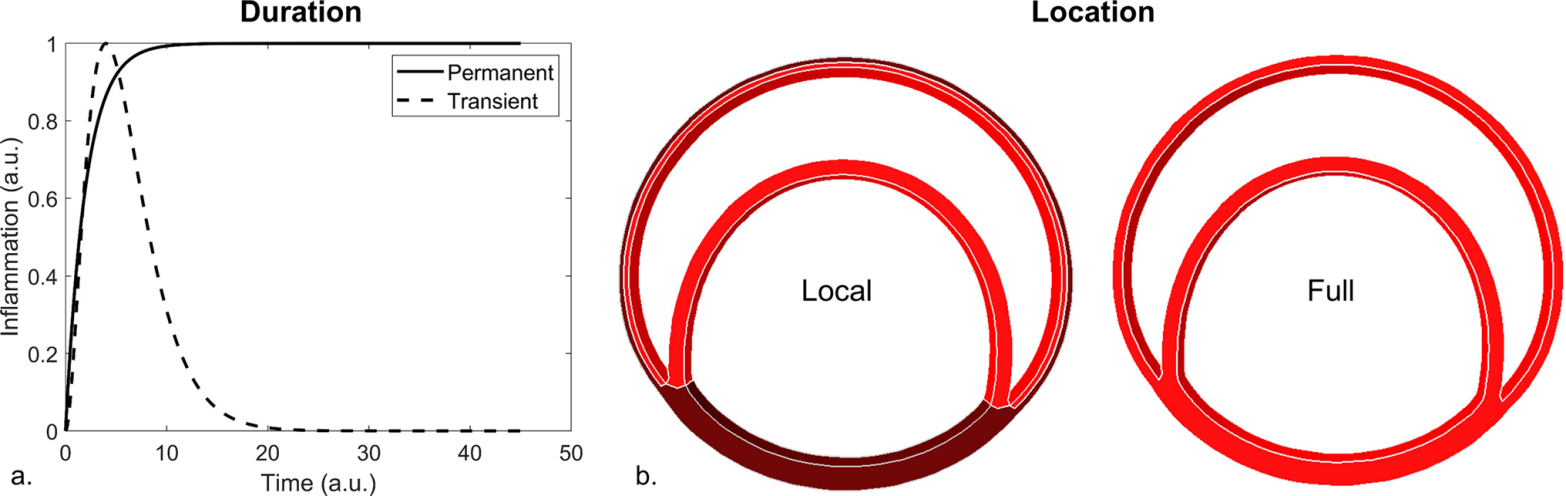
Schematic overview of the (**a**) duration, i.e. permanent or transient, and (**b**) location, i.e. full or local, of the inflammation patterns. For the (**b**) location, the area of inflammation is indicated in red

As inflammatory cells have been observed in the dissected region, inflammation might be involved in the process of the dissected membrane thickening (He et al. 2006; Luo et al. 2009; Wang et al. 2020). In total, four inflammation patterns were considered based on the duration and location of the inflammation. As indications have been found for a transient and permanent effect of inflammation on the dissected membrane (Peterss et al. 2016; Xu and Burke 2013), a transient and permanent inflammation process was studied similar to Maes et al. (2023) and Drews et al. (2020). The transient inflammatory reaction (Fig. 2a), as a consequence of the dissection of the aortic wall, was defined as

9 $${\Gamma }_{t}\left(s\right)= {\left(\frac{\delta s}{\beta -1}\right)}^{\beta -1}{\mathrm{e}}^{-\delta s+\beta -1},$$

with *δ* and $\beta$ being shape parameters that determine the location and extent of the inflammation peak and *s* being the time expressed in days. The second pattern implies a permanent inflammatory reaction triggered by the dissection, which is in correspondence to the observations of Peterss et al. (2016), where no significant change in degree of inflammation of the dissected membrane was found between the acute and chronic phase (Fig. 2a). The permanent inflammation function is described as

10 $${\Gamma }_{p}\left(s\right)= 1-{\mathrm{e}}^{-\delta s}$$

with *δ* being the shape parameter that defines how fast the inflammatory process arises after occurrence of the dissection and *s* being again the time expressed in days.

Next to parameters *δ* and $\beta$, three gain factors were defined which impose the extent to which the inflammation-mediated growth and remodeling of collagen and elastin takes place. Indeed, ${k}_{\Gamma +}^{c}$, ${k}_{\Gamma -}^{c}$ and ${k}_{\Gamma -}^{e}$, respectively, represent the extent of the collagen production, collagen degradation and elastin degradation as a consequence of the inflammatory reaction. Adding the effect of this inflammation to the stress-mediated growth and remodeling Eqs. (7) and (8) leads to

11 $${\dot{\rho }}_{+}^{c,f}\left(s\right)= \frac{{\rho }^{c,f}(s)}{{T}^{c}} \left(1+ {k}_{\Gamma +}^{c}\Gamma (s) + {k}_{\sigma }^{c}\frac{{\sigma }^{c,f}(s)- {\sigma }_{h}^{c,f}}{{\sigma }_{h}^{c,f}}\right)$$

12 $${\dot{\rho }}_{-}^{c,f}\left(s\right)= -\frac{{\rho }^{c,f}(s)}{{T}^{c}} \left(1+ {k}_{\Gamma -}^{c}\Gamma (s)\right)$$

13 $${\dot{\rho }}_{-}^{e}\left(s\right)= {-{1/3}{\rho }^{e}(s)k}_{\Gamma -}^{e}\Gamma \left(s\right).$$

Here, $\Gamma$ can be replaced by ${\Gamma }_{t}$ or ${\Gamma }_{p}$, depending on the considered inflammation duration. The location of the inflammation was either applied to the full wall, i.e. the complete medial and adventitial layer, or to the media that surrounds the false lumen, i.e. the dissected membrane and the media of the remaining wall, which is referred to as local inflammation (Fig. 2b). The regions which are not subjected to inflammation are still affected by the stress-mediated growth and remodeling and Eqs. (7) and (8), thus, apply.

#### Implications of the growth and remodeling algorithm

The applied homogenized constrained mixture algorithm has been discussed before (Maes et al. 2023), but it is worth to touch upon some implications of the considered dissected wall growth and remodeling. The modeled stress-mediated growth and remodeling depends on the difference between the current and the homeostatic collagen fiber stress, ${\sigma }^{{c},{f}}({s})$ and ${\sigma }_{h}^{{c},{f}}$, respectively, where collagen production is triggered in case of increased fiber stress. This is expected to happen in the remaining wall of the dissected aorta, as a thinner wall has to withstand the same pressure. Conversely, the production of fibers decreases if their stress level is below the homeostatic state, which is the case in the acute dissected membrane, where the delamination corresponds to the release of the stresses in the tissue and, consequently, in the collagen fibers.

Besides, note that the combination of stress- and inflammation-mediated remodeling implies that the collagen fibers remodel, even though their stress state equals the homeostatic one. In this case, the remodeling is triggered by the difference in inflammation-related production, ${k}_{\Gamma +}^{c}$, and degradation, ${k}_{\Gamma -}^{c}$. The concept that a severe inflammatory response might cause maladaptation and trigger the arterial wall to deviate from its homeostatic state was introduced by Latorre and Humphrey (2018), where it was applied in a constrained mixture model in the framework of hypertension.

### Parametric study of growth and remodeling parameters

#### Input parameters and sampling

As no clear parameter values for the stress- and inflammation-mediated growth and remodeling parameters (${k}_{\sigma }^{c}$, ${k}_{\Gamma +}^{c}$, ${k}_{\Gamma -}^{c}$, ${k}_{\Gamma -}^{e}$, *δ* and $\beta$ for transient inflammation and ${k}_{\sigma }^{c}$, ${k}_{\Gamma +}^{c}$, ${k}_{\Gamma -}^{c}$, ${k}_{\Gamma -}^{e}$ and *δ* for permanent inflammation) have been established in the framework of aortic dissections, previously reported values were used to define ranges with potential parameters (Table 1) (Braeu et al. 2017; Brandstaeter et al. 2021; Cyron et al. 2014; Maes et al. 2023; Mousavi et al. 2019). The resulting parameter space was sampled using a Latin hypercube with 1000 samples. Due to the different dimensions of the transient and permanent inflammation pattern, a different Latin hypercube sampling was required for both cases. The same Latin hypercube was, however, used for the local and full inflammation pattern. For all input parameters, a uniform probability distribution was assumed. For each Latin hypercube sample, a finite element analysis was performed and the success rate was assessed for the four inflammation patterns. Moreover, the range of the individual growth and remodeling parameters that resulted in a converged solution was determined.

**Table 1:** Overview of the applied parameter ranges for the Latin hypercube sampling of the transient and permanent inflammation pattern, based on published data on parameters used in the homogenized constrained mixture theory. The minimal (min) and maximal (max) values of the published ranges are reported (Braeu et al. 2017; Brandstaeter et al. 2021; Maes et al. 2023; Mousavi et al. 2019)

Parameter	Transient inflammation [min; max]	Permanent inflammation [min; max]
${k}_{\sigma }^{c}$(−)	[0.00; 0.42]	[0.00; 0.42]
${k}_{\Gamma +}^{c}$(−)	[1.74; 24.9]	[1.74; 24.9]
${k}_{\Gamma -}^{c}$(−)	[1.41; 20.7]	[1.41; 20.7]
${k}_{\Gamma -}^{e}$(step^−1^)	[0.000; 0.0707]	[0.000; 0.0707]
*δ* (days^−1^)	[0.0151; 0.478]	[2.77; 17.0]
$\beta$(−)	[1.10; 4.64]	NA

#### Output parameters

In correspondence to the definition of the acute, subacute and chronic dissection phase of Peterss et al. (2016), the thickness of the dissected membrane was considered at 0, 15, 42 and 90 days. Note that the subacute phase is commonly defined to start after 14 days, but that day 15 is considered since a time step of 3 days was chosen (Dake et al. 2013; Peterss et al. 2016). The thickness was determined by extracting the coordinates of the circumferentially central nodes at the true and false lumen side of the dissected membrane, throughout the axial direction, for each of the considered time points (Fig. 1b). Consequently, the thickening rates corresponding to the three phases were determined in case of 30 converged growth and remodeling time steps in the finite element analysis. The thickening rates, *R*_*T*_, of the dissected membrane were calculated as

14 $${R}_{T}= \frac{{T}_{DM}^{i}- {T}_{DM}^{j}}{i-j},$$

where ${T}_{DM}^{i}$ and ${T}_{DM}^{j}$ represent the thickness of the dissected membrane at day *i* and *j* (with *i* > *j*), respectively. Moreover, it was assessed which thickening rates agreed to the ranges as reported in Peterss et al. (2016). To account for the measurement uncertainty in the reported thickening measurements, additional uncertainty was added to the confidence intervals. A CT scan with a pixelsize of 1 mm was assumed, which leads to a maximal uncertainty of 2 mm per thickness measurement. As the thickening is the result of two thickness measurements, a total uncertainty of 4 mm was added to the lower and upper boundary of the reported confidence intervals. This resulted in an extended clinical range of [−3.50; 6.09] mm/year in the acute, [−4.13; 5.09] mm/year in the subacute and [−3.99; 4.03] mm/year in the chronic phase. In this respect, it was assessed which parameter values of the individual growth and remodeling parameters resulted in thickening rates within these extended clinical ranges.

The total diameter expansion of the aorta as a consequence of the dissection was assessed by calculating the cross-sectional distance of the total intraluminal space at day 0, 15, 42 and 90 (Fig. 1d). Moreover, the false and true lumen size were measured along this line as the distance between the inner side of the aortic wall and the middle of the dissected membrane. In this way, the sum of the true and false lumen sizes is by definition equal to the total diameter. As clinical data of the diameter expansion are often established based on follow-up data of multiple years, the expansion rate was estimated by extrapolating the diameter expansion using the growth rate in the chronic phase up to a duration of 365 days (Miyahara et al. 2011; Sueyoshi et al. 2009; Tolenaar et al. 2013; Trimarchi et al. 2013). The diameter expansion rate is, therefore, calculated as the sum of the modeled diameter expansion between day 0 and day 90 and the estimated diameter expansion from day 90 until day 365, by extrapolating the chronic expansion rate. In this way, the expansion rate, ${R}_{e}^{n}$, is represented in mm/year and expressed as

15 $${R}_{e}^{n}=\left({D}_{n}^{90}-{D}_{n}^{0}\right)+ \frac{{D}_{n}^{90}-{D}_{n}^{42}}{90-42}\left(365-90\right),$$

with superscript *n* referring to the respective total (tot), false lumen (FL) and true lumen (TL) and ${D}_{n}^{i}$ indicating the corresponding diameter at time *i*, which is indicated in days. The change in volume of the dissected membrane, i.e. the combined volume of the elastin and collagen, over time was assessed as well.

In order to consider the change in the dissected membrane microstructure, the evolution of the collagen and elastin content, expressed relative to the initial volume, was analyzed at day 15, 42 and 90, similar to the thickening rates. At day 0, the collagen and elastin content corresponds to the predefined fractions as indicated in Sect. 2.1.2. The collagen and elastin content was determined as the average of the content of each element of the dissected membrane, weighted by the corresponding volume.

## Results

### Parametric study of growth and remodeling parameters

For the full and local permanent inflammation patterns, convergence until the chronic phase (90 days) is reached for only 3 (0.3%) and 1 (0.1%) of the 1000 samples, respectively. The transient inflammation patterns result in higher success rates of 146 (14.6%) cases for the full and 88 (8.8%) for the local inflammation. Slightly lower success rates are, thus, found for the local compared to the full inflammation, irrespective of the considered duration. Due to the very limited convergence of the permanent inflammation patterns, the reported results are focused on the transient inflammation patterns. Some additional results regarding the permanent inflammation are, nevertheless, shown in appendix 2.

**Fig. 3:**
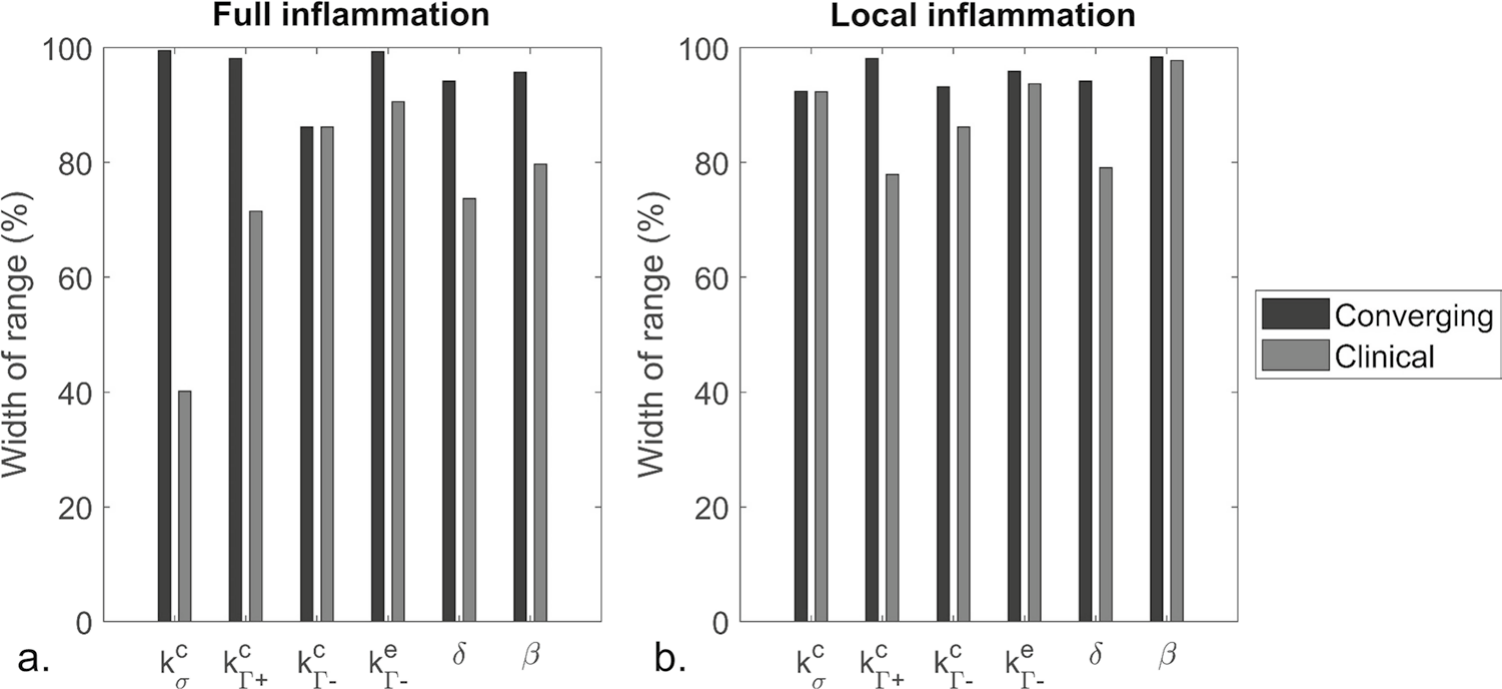
Overview of the width of the parameter range of the simulated samples that fulfilled the convergence criteria of the simulation during 90 days of growth and remodeling (converging) and resulted in thickening rates within the extended clinical range (clinical) for the full and the local transient inflammation pattern. The width is expressed as percentage of the initial width of the parameter range

An overview of the individual growth and remodeling parameter ranges that lead to convergence for growth and remodeling with transient inflammation over 90 days is given in Fig. 3, for all combinations that resulted in a converged solution and the selection of combinations with thickening rates within the extended clinical range (Sect. 3.2). Overall, a larger reduction in parameter ranges is established for the full inflammation, compared to the local one. When combining the relative reduced individual parameter widths that result in convergence over 90 days, the total parameter space is reduced to 75.3% and 75.1% of the initial parameter space for the full and local transient inflammation pattern, respectively. When further refining the parameter space to those parameter ranges that lead to thickening rates within the extended clinical ranges, the parameter space related to the full and local inflammation pattern reduces to 13.2% and 44.9% of the initial space.

### Example cases

**Fig. 4:**
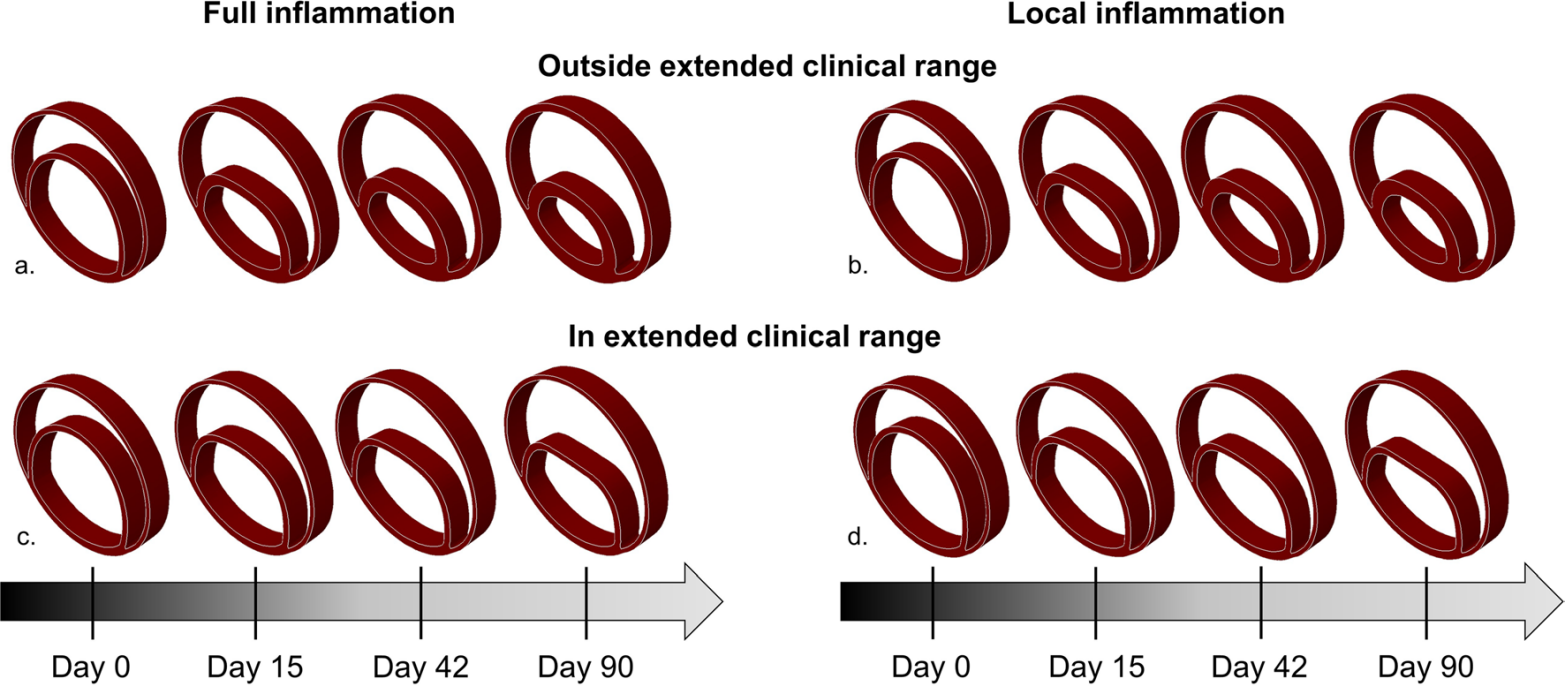
Examples of the predicted changes in geometry over time due to the growth and remodeling for samples with transient inflammation that is applied (**a**, **c**) over the full geometry and (**b**, **d**) locally around the false lumen, which result in thickening rates (**a**, **b**) outside and (**c**, **d**) within the extended clinical range

**Fig. 5:**
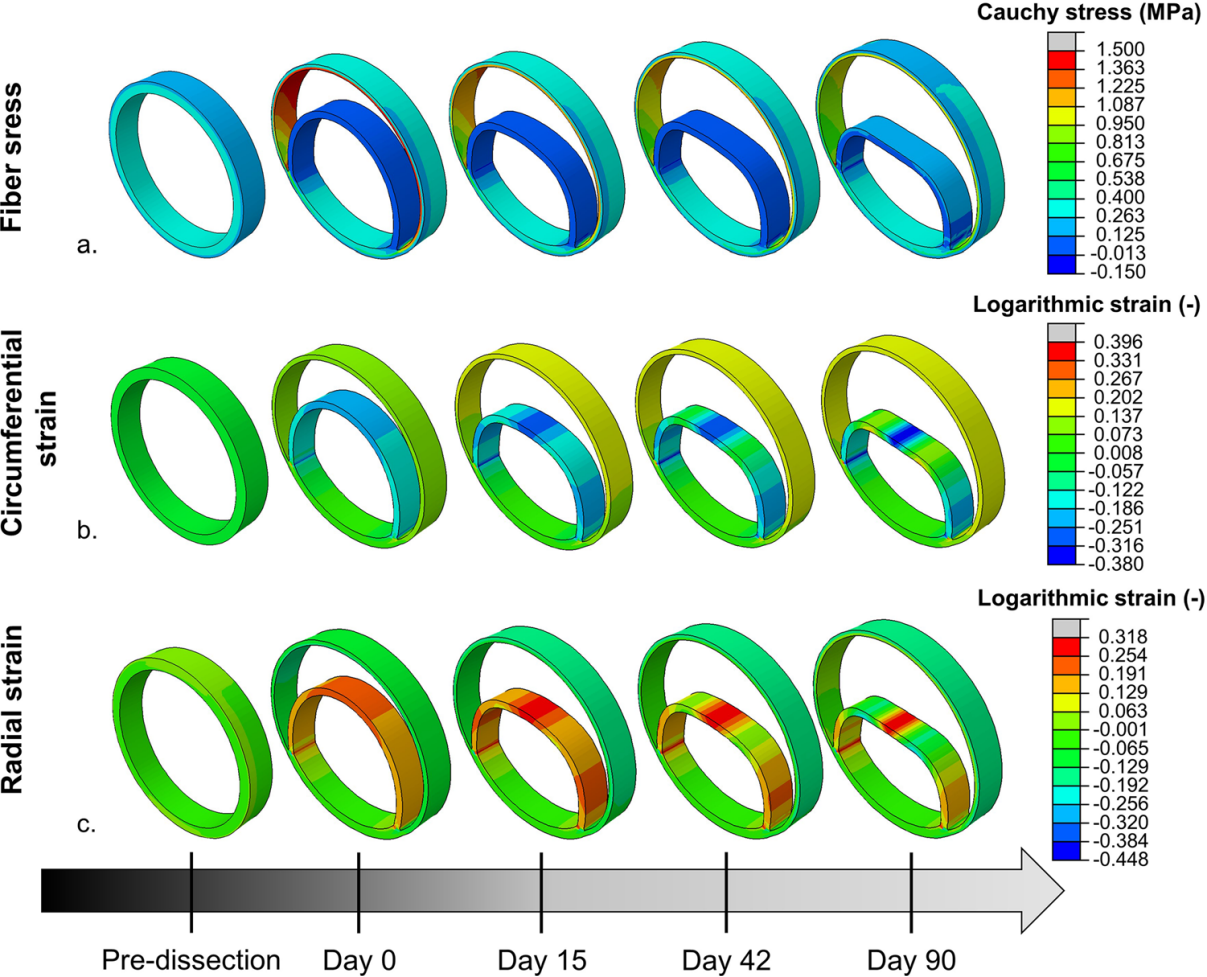
Evolution of the (**a**) fiber stress and (**b**) circumferential and (**c**) radial strain over time for an example sample with local and transient inflammation. The stress is expressed as the fiber Cauchy stress, while the circumferential and radial logarithmic strain are indicated to represent the deformation. The fiber stress is illustrated for one fiber family in the medial and one in the adventitial layer, which is representative for the second fiber family too. The radial and circumferential direction is defined according to the pre-dissected configuration

**Fig. 6:**
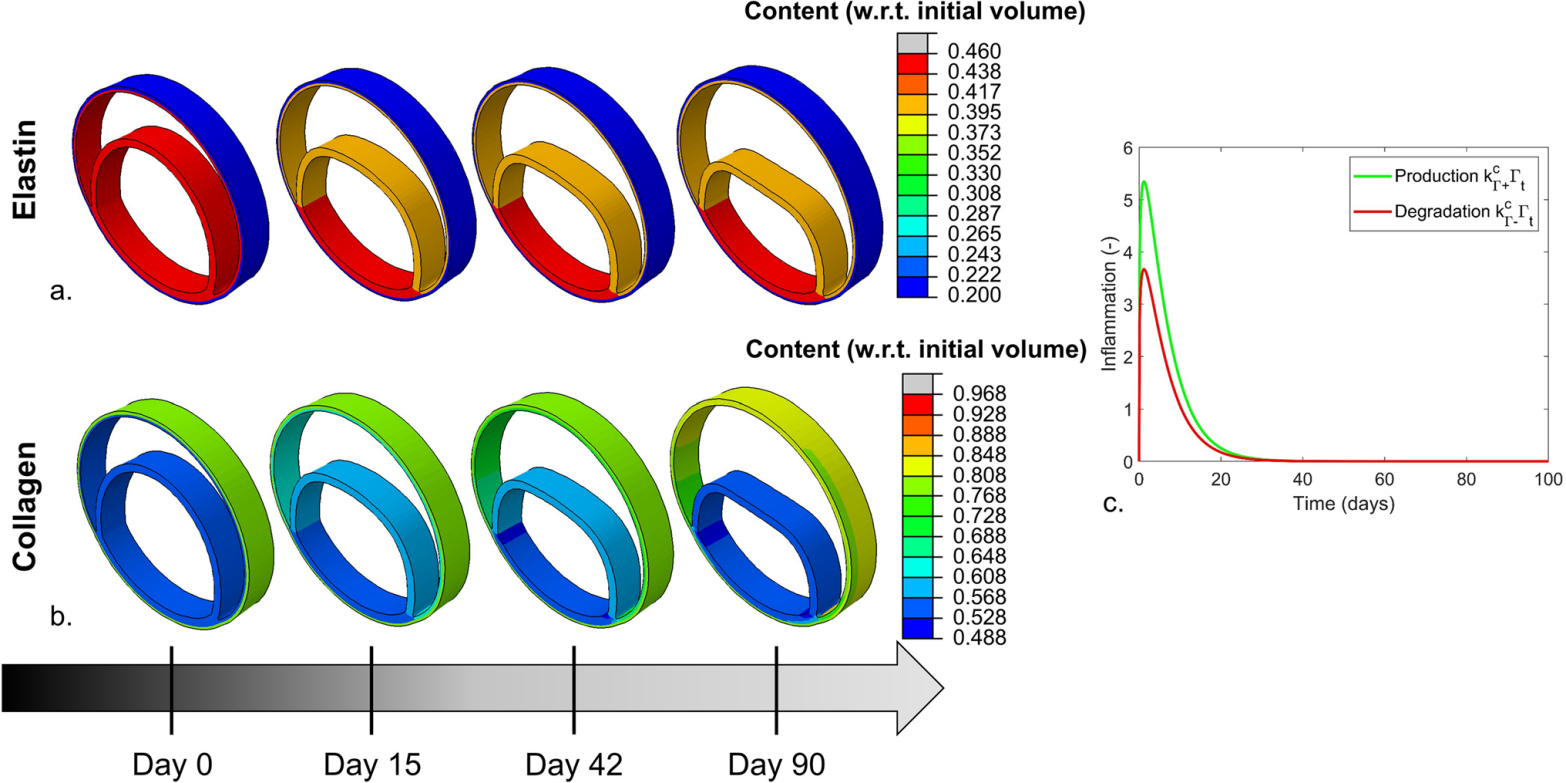
Evolution of the (**a**) elastin and (**b**) collagen content over time for an example sample with a local and transient inflammation pattern as illustrated in (**c**). The content is expressed as fraction of the initial volume

The deformed configurations at day 0, 15, 42 and 90 of four illustrative transient inflammatory example cases are illustrated in Fig. 4 and in appendix 3, while the change of their stresses and deformations over time is included as animation in the online supplementary material. For the example case with transient, local inflammation and thickness rates within the extended clinical range, Figs. 5 and 6, respectively, present the change in strain and fiber stress and the change in collagen and elastin content in the dissected wall over time. The corresponding growth and remodeling parameters of this example are ${k}_{\sigma }^{c}$ = 0.130, ${k}_{\Gamma +}^{c}$= 5.34, ${k}_{\Gamma -}^{c}$= 3.67, ${k}_{\Gamma -}^{e}$= 0.0533 step^−1^, $\delta$ = 0.198 day^−1^ and $\beta$ = 1.24.

As a consequence of the dissection, the fiber stress in the remaining wall initially increases, to decrease again during the growth and remodeling process. The opposite effect is seen for the dissected membrane. The elastin and collagen content only adjusts once the growth and remodeling process starts. Therefore, Fig. 6 does not include the pre-dissection configuration. The growth and remodeling process induces a sudden decrease in the elastin content between day 0 and day 15 in the portion of the media that is subjected to inflammation. Afterwards, the elastin content remains, approximately, constant. The content of collagen initially decreases in the dissected membrane, to increase again from day 42 onwards, while an overall increase in collagen content is observed in the adventitia of the remaining wall.

### Thickening rates of the dissected membrane

**Fig. 7:**
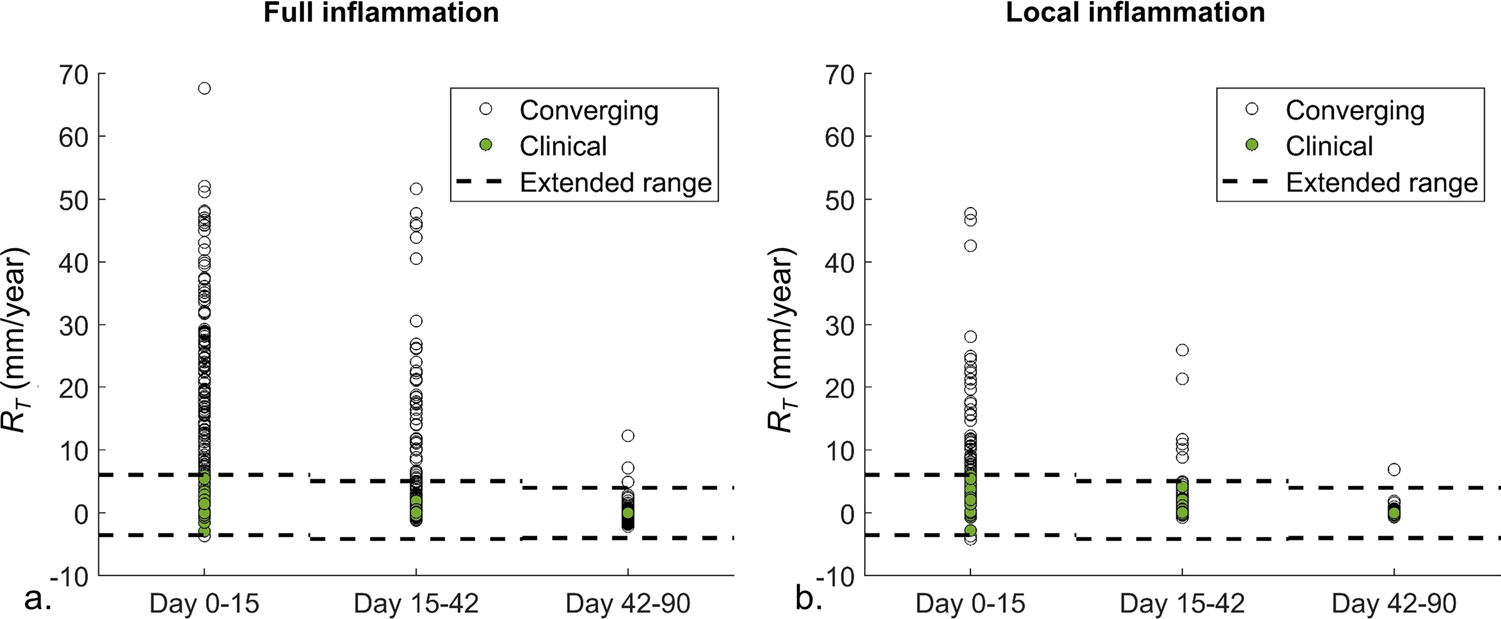
Resulting thickening rates, *R*_*T*_, of the dissected membrane for the (**a**) full and (**b**) local transient inflammation pattern, for the acute (Day 0–15), subacute (Day 15–42) and chronic (Day 42–90) phase. The rates corresponding to converging simulations are indicated with black circles. The points corresponding to converged simulations that lead to thickening rates within the extended clinical range are filled in green. The upper and lower boundaries of the extended clinical range for the three phases are shown with a dashed line

Figure 7 shows the dissected membrane thickening over time for the converged samples of the transient inflammatory cases. A decreasing thickening rate over time is found for the local and full transient inflammation pattern, where, respectively, 24 (2.4%) and 38 (3.8%) samples result in rates within the proposed extended clinical ranges for the acute, subacute and chronic phase. An overview of the corresponding growth and remodeling parameters is given in appendix 4. In contrast, the converged samples with permanent inflammation indicate an increasing trend in thickening rate over time, for the local as well as the full inflammation pattern (appendix 2).

### Diameter expansion and volume change

Over the considered 90 days of soft tissue growth and remodeling, the total diameter increases for all parameter combinations within the extended clinical thickening range and a median total diameter expansion rate of 5.09 mm/year and 4.71 mm/year is observed for the full and local inflammation pattern, respectively (Table 2). The false lumen size increases with a median rate of 19.6 mm/year for full and 19.7 mm/year for local inflammation. The true lumen size, however, decreases over time at a respective median rate of 15.0 mm/year and 14.8 mm/year for the full and local inflammation pattern. The volume of the dissected membrane at 90 days varies from 84 to 106% of the initial volume for the full and from 84 to 111% for the local inflammation pattern.

**Table 2:** Expansion rate of the total diameter (${R}_{e}^{tot}$), the true lumen (${R}_{e}^{TL}$) and the false lumen (${R}_{e}^{FL}$), expressed as mm/year, based on extrapolation of the expansion rate in the chronic phase up to a period of 365 days, for the full and local transient inflammation patterns that resulted in thickening rates within the extended clinical range. The median and the minimal (min) and maximal (max) expansion rate are indicated

Expansion rate	Full inflammation median [min; max]	Local inflammation median [min; max]
${R}_{e}^{tot}$(mm/year)	5.09 [2.57; 7.69]	4.71 [2.59; 7.76]
${R}_{e}^{TL}$(mm/year)	−15.0 [−20.8; −13.7]	−14.8 [−21.4; −13.4]
${R}_{e}^{FL}$(mm/year)	19.6 [16.7; 26.2]	19.7 [16.3; 26.8]

### Dissected membrane microstructure

**Fig. 8:**
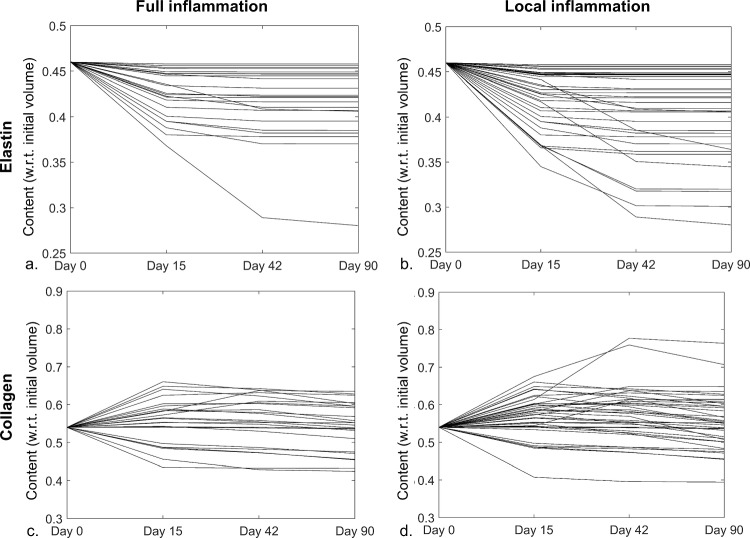
Overview of the (**a**, **b**) elastin and (**c**, **d**) collagen content of the dissected membrane for the (**a**, **c**) full and (**b**, **d**) local inflammation pattern, for the parameter combinations that lead to thickening rates within the extend clinical range. The content is expressed relative to the initial volume of the dissected membrane

An overview of the elastin and collagen content at day 90 is shown in Fig. 8 for the parameter combinations resulting in thickening rates within the extended clinical ranges. For both inflammation patterns, the amount of elastin remains similar or decreases, which results at day 90 in an elastin content between 28.0% and 45.8% of the initial volume (elastin content at day 0: 46%). The trend in collagen content is variable. At day 90, the collagen content of the full inflammation pattern ranges from 42.5 to 63.5% of the initial volume (collagen content at day 0: 54%) and the respective minimal and maximal collagen content as result of the local inflammation pattern varies from 39.5% to 76.4% of the initial volume. This indicates that both decreasing and increasing collagen contents are observed.

## Discussion

The current study aims to evaluate whether existing growth and remodeling algorithms, fed with parameters from literature, allow the reproduction of the soft tissue growth and remodeling in aortic dissections, with a particular focus on the thickening and change in microstructure of the dissected membrane. A slice model of an acute dissection with an anisotropic hyperelastic material that accounts for the deposition stretches was used as starting point and a homogenized constrained mixture model was applied to represent a period of 90 days. The model included collagen production as a consequence of deviations from the homeostatic stress state as well as collagen and elastin degradation and/or production triggered by inflammation. Four inflammatory response patterns were considered, depending on the location and duration of the inflammation.

The overall success rate is limited to 15% for the considered inflammation patterns, which indicates that only particular growth and remodeling parameters are compatible. In this respect, the difference between the inflammation-mediated collagen production and degradation is hypothesized to be an important factor as only about 60% of the total ${k}_{\Gamma +}^{c}-{k}_{\Gamma -}^{c}$ range is able to reach convergence for transient inflammation, which further decreases to 4% for permanent inflammation (Table 1). Introducing a dependency between both parameters in the sampling is expected to enhance the success rate of the model. Furthermore, a clear difference in success rate between transient and permanent inflammation is observed. Indeed, the transient inflammation results in success rates of 9% to 15%, while a maximal rate of only 0.3% is found for the permanent inflammation. Furthermore, the obtained thickening rates for the few converging samples with permanent inflammation show an increasing trend (appendix 2), which would by definition result in increasingly thicker membranes until non-physiological thicknesses would be obtained. This persistent thickening is also hypothesized to be the main reason for the very limited convergence. Consequently, the permanent inflammation pattern is unrealistic and not able to reproduce the clinical observations of dissected membrane thickening. This suggests that transient inflammation, rather than permanent inflammation, takes place after dissection of the aorta. Although Peterss et al. (2016) reported no significant difference in the presence of inflammation between dissections in the acute and chronic phase, the transient pattern is in line with the findings of Xu and Burke (2013), who reported that inflammation mainly occurred in the first week after dissection.

It is important to highlight that this study is not aimed towards revealing the ground-truth growth and remodeling mechanisms and corresponding parameters in aortic dissections. Rather, this study investigates up to which extent the clinical observations of dissection patients can be reproduced based on the existing soft tissue growth and remodeling knowledge. As such, the ability to reproduce clinical trends does not necessarily imply that the model captures the physiological reality. It only supports the assumptions that transient inflammation potentially plays an essential role in the soft tissue growth and remodeling of aortic dissections.

The resulting median total diameter expansion rates are 5.09 mm/year and 4.71 mm/year, respectively, for the full and local transient inflammation (Table 2). Experimentally, mean and median total diameter expansion rates of 2.10 mm/year up to 5.28 mm/year were established for type B dissections with a patent false lumen (Miyahara et al. 2011; Sueyoshi et al. 2009; Tolenaar et al. 2013; Trimarchi et al. 2013). The model, thus, predicts expansion rates that are in the upper part, but still within the range, of the available experimental data. The slight increase in predicted expansion rate, compared to the measurements, might be explained by the limited time frame of the modeled results. Despite the fact that only the chronic phase was used to extrapolate the diameter expansion to a duration of one year (Eq. (15)), experimental expansion rates were calculated over longer time frames, with mean and median follow-ups ranging from 19.5 to 48.9 months, which is expected to result in a lower average expansion rate (Miyahara et al. 2011; Sueyoshi et al. 2009; Tolenaar et al. 2013; Trimarchi et al. 2013). Indeed, the largest expansion rates have been observed in the first days after the dissection, whereas lower mean expansion rates of 1.2 mm/year to 1.5 mm/year are reported in the chronic phase (Berezowski et al. 2022; Kelly et al. 2007). Moreover, slight differences in the methodology to obtain the total diameter, e.g. considering the short-axis diameter, might lead to a discrepancy between the experimental and the modeled results too (Sueyoshi et al. 2009). Taking this into account, the model predictions reasonably approach the existing measurements.

Only few data are available on the evolution of the true and false lumen size, but an increase of the false lumen size over time, both in absolute and relative terms, has been observed, in line with the simulated data (Blount and Hagspiel 2009; Song et al. 2007). The extent to which the false lumen size increases, however, differs. While the current results show a median expansion rate of 19.6–19.7 mm/year in absolute terms, which corresponds to a relative increase from 25% to about 49% of the corresponding total diameter, a mean false lumen growth rate of 6.52 mm/year and a relative increase up to 12% of the total diameter was found (Blount and Hagspiel 2009; Song et al. 2007). Even lower growth rates were established when considering growth from the chronic phase on (Kelly et al. 2007). Regarding the true lumen, a slight increase in absolute size was reported, with growth rates of 0.8 mm/year to 1.0 mm/year, both for measurements starting at the acute and chronic phase (Blount and Hagspiel 2009; Kelly et al. 2007). A strong decrease, with median rates up to -15 mm/year, is, on the contrary, found in the current study. It is hypothesized that the distinction between the resulting and reported true and false lumen growth rates is related to the use of a slice model, where the connection of the dissected membrane to the healthy aortic wall is limited and the movement of the dissected membrane, thus, less restricted compared to a patient-specific dissected aorta (Fig. 1). Moreover, the large circumferential size of the false lumen included in the model might play a role. Indeed, a false lumen angle of 245°, obtained based on a patient-specific CT scan, is at the higher range of the physiological values (Brunet et al. 2023; Logghe et al. 2021). Next to a strong decrease in true lumen diameter, a flattening of the true lumen was observed. To the authors’ knowledge, experimental data which could confirm or contradict this observed trend are currently lacking and results are to be interpreted keeping in mind the explorative computational character of the study.

In this respect, it is noteworthy that the implemented material model assumes that the collagen fibers do not contribute to load bearing in compression. Doubts have, however, been raised regarding this assumptions (Horgan and Murphy 2020). If collagen fibers would provide resistance against deformation in compression, decreased displacements of the dissected membrane and changes in true and false lumen size are expected over time. This might have some impact on the resulting thickening rates, the stress-mediated (decrease in) collagen production and, consequently, the collagen content of the dissected membrane. While determining the effect of this assumption on the dissected membrane would be of interest, no major effect on the total diameter expansion rate is expected. The assumption is, therefore, expected to affect the selection of material parameters that lead to thickening rates within the clinical extended range, rather than the pathophysiological relevance of the presented model.

The evolution of the false lumen, true lumen and total diameter was found to be similar for the full and local transient inflammation pattern (Table 2). On the one hand, the change in true and false lumen diameter is generally dictated by the deformation of the dissected membrane. The total diameter, on the other hand, is mainly impacted by the deformation of the remaining wall as a consequence of the increased load due to the dissection. As the medial collagen fibers are more circumferentially oriented compared to the adventitial ones, the medial tissue strength provides the highest resistance against the deformation, which takes place in the circumferential direction as the axial stretch is fixed. The medial layer that surrounds the false lumen, thus, largely determines the overall change in geometry, while the adventitial layer and the media of the true lumen wall play a limited role. As this tissue portion is not differently affected by the local and full inflammation pattern, no major effects in the overall range of diameters are observed.

Interestingly, the overall modeled changes in microstructure correspond to the experimental observations. Indeed, the model indicates a decrease in functional elastin content over time, induced by the inflammation, which agrees with the presence of fragmented elastin in the dissected membrane (Fig. 8) (Sariola et al. 1986; Schlatmann and Becker 1977; Wang et al. 2006). While fibrosis, indicating an abundant presence of collagen, has been experimentally observed in the dissected membrane, the collagen content of the considered samples either increased or decreased (Schlatmann and Becker 1977; Wang et al. 2006). In this respect, it is worth noting that no constraints on the relation between ${k}_{\Gamma -}^{c}$ and ${k}_{\Gamma +}^{c}$ were applied, which implies that for some samples a higher inflammation-mediated collagen degradation than production was imposed. These simulations, naturally, lead to a net decrease in collagen. No requirements regarding the balance between the stress- and inflammation-mediated growth and remodeling were defined either. Indeed, the collagen production is expected to be purely attributed to the inflammation as the dissected membrane is in a quasi-stress-free state in the acute phase, which will impose a negative stress-mediated collagen production that decreases the overall collagen production. If ${k}_{\Gamma +}^{c}$ was only slightly higher than ${k}_{\Gamma -}^{c}$, a decrease in collagen content was obtained in case the corresponding ${k}_{\sigma }^{c}$ was relatively large, in particular when the inflammatory response was restricted to a limited time frame. This is also illustrated by two examples shown in appendix 5.

A slight difference between the local and full inflammation pattern was found in this respect. In case of local inflammation, 22 of the 38 samples that lead to a thickening rate within the extended clinical range resulted in the expected increased collagen content at day 90. For full inflammation, an increasing collagen content was observed for only 13 of the 24 samples within the extended clinical thickening range. Despite the limited differences between the full and local inflammation pattern, the presence of less samples with a decreasing collagen content with local inflammation might imply that the local inflammation is slightly more appropriate to represent the physiological process.

Although an increase in collagen and a decrease in elastin in the dissected membrane correspond to the reported data, a quantitative comparison with published experiments is not straightforward (Sariola et al. 1986; Schlatmann and Becker 1977; Wang et al. 2006). Indeed, the model considers the functional elastin and collagen, while e.g. fragmented elastin might be part of the experimental staining, and thus the published area fractions. Moreover, the model only accounts for elastin and collagen, whereas other constituents are intrinsically included in the total soft tissue area determined in experiments. Thus, the total area or volume obtained from the model and experiments might differ. Furthermore, it is not always unambiguously indicated whether the results are compared to control samples in absolute amounts or with respect to the corresponding total soft tissue area/volume (Wang et al. 2006).

### Limitations

The presented model approaches the soft tissue growth and remodeling in aortic dissections in terms of thickening rates, total diameter expansion rates and changes in elastin and collagen, but it does not account for the presence of other constituents as smooth muscle cells or glycosaminoglycans. Glycosaminoglycans are, however, expected to play a role in the dissection initiation and progression (Ahmadzadeh et al. 2018; Humphrey 2013). Next, the model represents an idealized aortic dissection, without tears or healthy aortic wall, which is strongly simplified compared to a patient-specific geometry. Moreover, the obtained results are based on one set of HGO material parameters for the medial and adventitial layer and, thus, no fiber dispersion was included. The material parameters were selected based on a representative pulse wave velocity for a healthy middle-aged human. As differences in the behavior of healthy and (pre-)dissected wall tissue are expected (Deplano et al. 2019), the considered pulse wave velocity and the corresponding material parameters should be considered as an approximation of the acute dissected wall behavior. However, no representative information on the pulse wave velocity of pre-dissection or acute dissection patients was available, to the authors’ knowledge.

For the acute material behavior, area fractions were used to determine the relation between the same constituents of the medial and adventitial layer, on the one hand, and between the elastin and collagen fractions within each material layer, on the other hand. This option was chosen in order to represent the elastin and collagen content in a more intuitive manner, i.e. as fraction of a tissue portion of the considered layer that contains no other constituents than elastin and collagen. To confirm that the impact is limited to the representation of the constituent content, the example case shown in Figs. 5 and 6 was repeated with the amount of elastin and collagen provided in absolute terms (data not shown). The maximal elementwise difference in fiber stress was limited to 1% for the dissected wall after 90 days of growth and remodeling, when comparing the applied model to a model where the elastin and collagen content was expressed in absolute terms. This negligible difference is hypothesized to be the consequence of the rounding during the calculation of the relative fractions.

For the dissected membrane, no net load was assumed, to reduce the simulation time as convergence was more easily achieved. However, this assumption is an approximation as it neglects the difference in surface of both sides of the dissected membrane on which the pressure is applied. A comparison with and without an explicit pressure load on both surfaces of the dissected membrane indicated an average elementwise difference of 2% relative to the maximal fiber stress in the dissected membrane at the start of the growth and remodeling process (data not shown). This limited effect on the fiber stress of the dissected membrane, therefore, justifies the assumption that there is no net loading at the dissected membrane.

Furthermore, the current knowledge on the evolution of the dissected membrane thickness over time is limited. To the best of our knowledge, the only reference reporting quantitative thickening rates of the dissected membrane is Peterss et al. (2016), with thicknesses measured from CT scans, which imposes quite some uncertainty on the obtained ranges. This uncertainty was accounted for by including an additional range of 8 mm to the 95% confidence interval. This extended clinical range might, however, overestimate the true uncertainty of the CT measurement. Despite the potential overestimation, the predefined range allows the determination of a selection of growth and remodeling parameters that are able to approximate the experimental observations. This selection can be applied to guide the reduction of the total growth and remodeling parameter space to a subspace with a higher rate of potentially physiological parameter combinations, which are usable in computational models.

## Conclusion

An idealized model was developed to represent the dissected membrane thickening during the transition from acute to chronic dissection as a consequence of stress- and inflammation-mediated growth and remodeling. A parametric study of the growth and remodeling parameters was performed for multiple inflammation patterns that differed based on the applied location and duration. For a subset of the considered growth and remodeling parameter space, the transient inflammation was able to reproduce the experimentally observed trends of the thickening rates and the total diameter expansion rate. The changes in elastin and collagen content over time could be represented by the transient inflammation as well, in particular when it was locally applied around the false lumen. The developed model framework is, consequently, able to represent the clinically observed situation, while it also provides guidance to reduce the growth and remodeling parameter space.

### Electronic supplementary material

Below is the link to the electronic supplementary material.

Supplementary file1: Documentation (TXT 1 kb)

Supplementary file2: Transient - Full - Outside extended clinical range - Deform (MP4 545 kb)

Supplementary file3: Transient - Full - Outside extended clinical range - Displacement (MP4 763 kb)

Supplementary file4: Transient - Full - Outside extended clinical range - Stress (MP4 794 kb)

Supplementary file5: Transient - Full - Inside extended clinical range - Deform (MP4 547 kb)

Supplementary file6: Transient - Full - Inside extended clinical range - Displacement (MP4 764 kb)

Supplementary file7: Transient - Full - Inside extended clinical range - Stress (MP4 786 kb)

Supplementary file8: Transient - Local - Outside extended clinical range - Deform (MP4 545 kb)

Supplementary file9: Transient - Local - Outside extended clinical range - Displacement (MP4 743 kb)

Supplementary file10: Transient - Local - Outside extended clinical range - Stress (MP4 862 kb)

Supplementary file11: Transient - Local - Inside extended clinical range - Deform (MP4 548 kb)

Supplementary file12: Transient - Local - Inside extended clinical range - Displacement (MP4 762 kb)

Supplementary file13: Transient - Local - Outside extended clinical range - Stress (MP4 787 kb)

## Figures and Tables

**Fig. 9:**
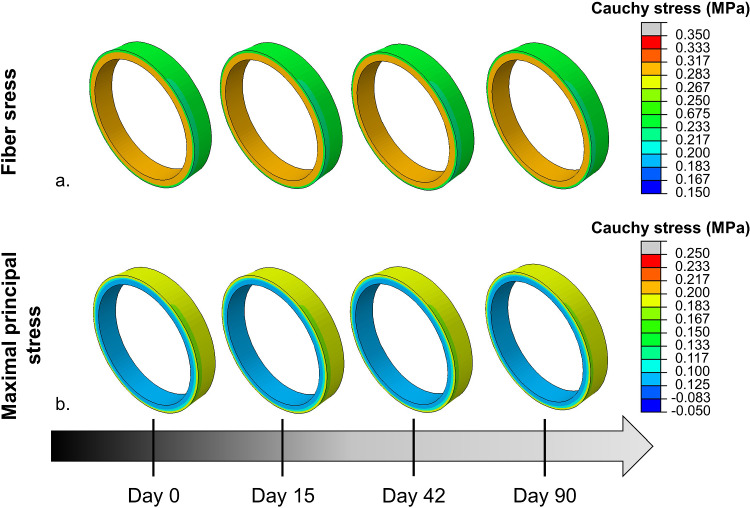
Evolution of the collagen fiber and the maximal principal Cauchy stress during stress-mediated growth and remodeling of a healthy aortic wall for an example sample, i.e. the same sample as indicated in Fig. 5 with ${k}_{\sigma }^{c}=0.130$. Note that the fiber stress is shown for one fiber family of the medial and adventitial layer, which is representative for the second family too

**Fig. 10:**
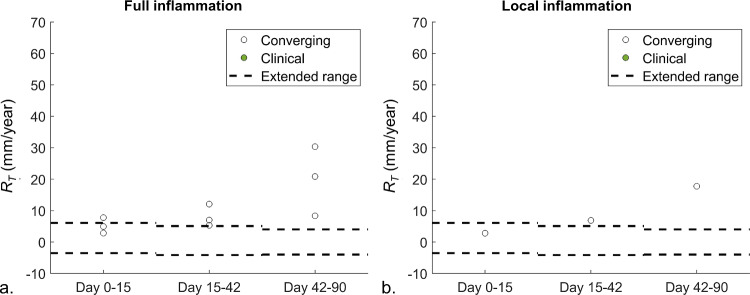
Resulting thickening rates, *R*_*T*_, of the dissected membrane for the (**a**) full and (**b**) local permanent inflammation pattern, for the acute (Day 0–15), subacute (Day 15–42) and chronic (Day 42–90) phase. The rates corresponding to converging simulations are indicated with black circles. The points corresponding to converged simulations that lead to thickening rates within the extended clinical range would be filled in green, but are not present for the permanent inflammation pattern. The upper and lower boundaries of the extended clinical range for the three phases are shown with a dashed line

**Fig. 11:**
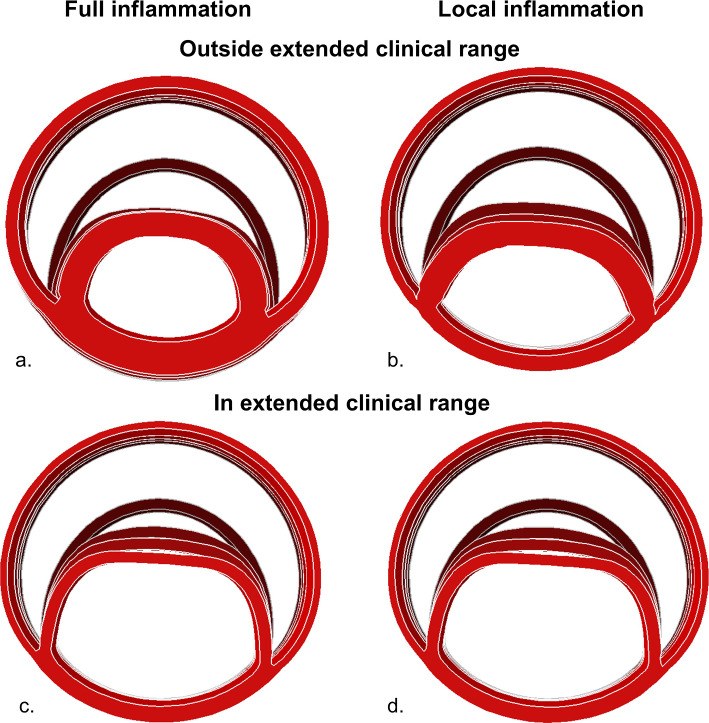
Front view of the predicted changes in geometry over time due to the growth and remodeling for samples with transient inflammation that is applied (**a**, **c**) over the full geometry and (**b**, **d**) locally at the false lumen, which result in thickening rates (**a**, **b**) outside and (**c**, **d**) within the extended clinical range. The presented samples correspond to those illustrated in Fig. 2. The deformed configurations at day 0, 15, 42 and 90 are shown in an increasingly bright shade of red

**Fig. 12:**
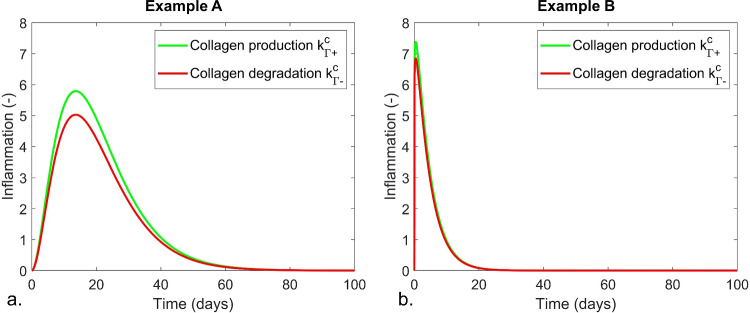
Overview of the transient inflammation pattern, ${\Gamma }_{\mathrm{t}}$, of (**a**) example A and (**b**) example B. The pattern is indicated for the inflammation-mediated collagen production, ${k}_{\Gamma +}^{c}$, and degradation, ${k}_{\Gamma -}^{c}$, in green and red, respectively

**Fig. 13:**
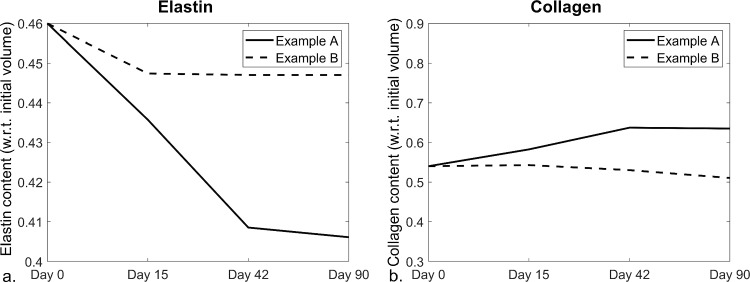
Evolution of the (**a**) elastin and (**b**) collagen content of both example samples. The evolution of example A and B is, respectively, indicated with a full and dashed line. Note that the constituent content was determined at day 0, 15, 42 and 90. The values in between were obtained via linear interpolation

## Appendix 1

### Stress-mediated growth and remodeling of a healthy aortic wall

See Fig. 9.

## Appendix 2

### Thickening rates for the permanent inflammation patterns

See Fig. 10.

## Appendix 3

### Time evolution of example cases with a transient inflammation pattern

See Fig. 11.

## Appendix 4

### Parameter combinations with clinically observed thickening rates

Tables 3 and 4.

**Table 3:** Growth and remodeling parameter combinations that result in thickening rates within the extended clinical range for the transient full inflammation pattern

${k}_{\sigma }^{c}$ (−)	${k}_{\Gamma +}^{c}$ (−)	${k}_{\Gamma -}^{c}$ (−)	${k}_{\Gamma -}^{e}$ (step^−1^)	*δ* (days^−1^)	$\beta$ (−)
0.0150	3.57	3.55	0.0403	0.349	3.23
0.0741	11.6	10.6	0.0476	0.390	1.82
0.0842	3.12	2.16	0.0145	0.253	1.37
0.0328	4.30	2.82	0.0165	0.326	3.09
0.0520	5.79	5.03	0.0147	0.145	2.97
0.130	5.34	3.67	0.0533	0.198	1.24
0.169	9.90	3.13	0.0135	0.343	1.28
0.0751	18.6	16.8	0.0555	0.215	1.16
0.0677	4.89	2.50	0.0671	0.454	3.24
0.000749	4.46	7.87	0.0384	0.357	1.86
0.0337	4.97	6.46	0.0230	0.296	3.86
0.00175	5.09	4.58	0.0155	0.301	1.35
0.0179	7.47	7.42	0.0462	0.171	1.52
0.0990	5.62	6.91	0.00875	0.406	1.87
0.0456	7.88	11.3	0.0367	0.460	1.41
0.168	3.83	2.96	0.00757	0.227	2.98
0.0548	2.83	1.66	0.0227	0.276	2.41
0.0460	2.01	1.46	0.0541	0.119	2.47
0.112	13.5	18.1	0.0197	0.450	1.21
0.0445	3.44	2.43	0.0420	0.339	3.93
0.123	5.11	6.97	0.00311	0.356	1.54
0.0896	2.32	2.00	0.0429	0.227	2.69
0.113	7.38	6.85	0.0217	0.249	1.11
0.134	16.4	7.16	0.0124	0.436	1.22

**Table 4:** Growth and remodeling parameter combinations that result in thickening rates within the extended clinical range for the transient local inflammation pattern

${k}_{\sigma }^{c}$ (−)	${k}_{\Gamma +}^{c}$ (−)	${k}_{\Gamma -}^{c}$ (−)	${k}_{\Gamma -}^{e}$ (step^−1^)	*δ* (days^−1^)	$\beta$ (−)
0.0150	3.57	3.55	0.0403	0.349	3.23
0.344	5.48	4.69	0.0173	0.0942	3.17
0.0741	11.6	10.6	0.0476	0.390	1.82
0.0842	3.12	2.16	0.0145	0.253	1.37
0.0328	4.30	2.82	0.0165	0.326	3.09
0.0520	5.79	5.03	0.0147	0.145	2.97
0.226	4.79	2.18	0.0643	0.293	4.57
0.130	5.34	3.67	0.0533	0.198	1.24
0.0868	6.06	4.80	0.0679	0.388	3.57
0.169	9.90	3.13	0.0135	0.343	1.28
0.0751	18.6	16.8	0.0555	0.215	1.16
0.0677	4.89	2.50	0.0671	0.454	3.24
0.290	11.9	9.53	0.0470	0.382	2.40
0.154	20.0	14.5	0.0342	0.394	1.18
0.00175	5.09	4.58	0.0155	0.301	1.35
0.00542	13.2	12.1	0.0628	0.271	4.33
0.0179	7.47	7.42	0.0462	0.171	1.52
0.224	16.1	13.4	0.0241	0.356	1.29
0.0990	5.62	6.91	0.00875	0.406	1.87
0.0456	7.88	11.3	0.0367	0.460	1.41
0.168	3.83	2.96	0.00757	0.227	2.98
0.0548	2.83	1.66	0.0227	0.276	2.41
0.0346	6.11	5.31	0.0693	0.195	2.77
0.0460	2.01	1.46	0.0541	0.119	2.47
0.129	17.3	17.1	0.0670	0.447	1.50
0.389	4.55	3.45	0.0206	0.243	3.49
0.112	13.5	18.1	0.0197	0.450	1.21
0.261	5.16	2.90	0.0691	0.457	4.24
0.163	5.50	3.77	0.0327	0.162	3.70
0.0445	3.44	2.43	0.0420	0.339	3.93
0.123	5.11	6.97	0.0031	0.356	1.54
0.0896	2.32	2.00	0.0429	0.227	2.69
0.0387	13.9	16.1	0.0472	0.393	1.55
0.00909	4.00	6.73	0.00740	0.370	3.50
0.113	7.38	6.85	0.0217	0.249	1.11
0.158	7.42	7.17	0.0104	0.447	3.20
0.239	4.81	4.09	0.00340	0.425	2.44
0.134	16.4	7.16	0.0124	0.436	1.22

## Appendix 5

### Evolution of elastin and collagen content of two example samples

The evolution of the elastin and collagen content is illustrated for two example samples with local transient inflammation, in particular differing by the timing and width of the peak of the inflammatory response (A: peak at day 14, width at 50% of the peak of 24 days; B: peak at day 1, width at 50% of the peak of 4 days). The parameters of both examples are indicated in Table

**Table 5:** Growth and remodeling parameters of example A and B

Example	${k}_{\sigma }^{c}$ (−)	${k}_{\Gamma +}^{c}$ (−)	${k}_{\Gamma -}^{c}$ (−)	${k}_{\Gamma -}^{e}$ (step^−1^)	*δ* (days^−1^)	$\beta$ (−)
A	0.0520	5.79	5.03	0.0147	0.145	2.97
B	0.113	7.38	6.85	0.0217	0.245	1.11

^5^ and the transient inflammation pattern is illustrated in Fig. 12. Note that the inflammation-mediated collagen production, ${k}_{\Gamma +}^{c}$, is higher than the degradation, ${k}_{\Gamma -}^{c}$, in both examples. The peak of the inflammation pattern of example A extends, however, over a larger time frame compared to example B.

The resulting evolution in elastin and collagen content of the dissected membrane is shown in Fig. 13, where the content of elastin decreases for respective examples A and B to 40.6% and 44.8%. For collagen, the content increases to 63.5% for example A, while a decrease to 51.0% was found for example B. The reason for the overall decrease in collagen content in case B is attributed to the short period of inflammation in combination with the rather large decrease stress-mediated collagen production.

The discussed examples both have a transient inflammation pattern. Note, however, that the evolution in dissected membrane microstructure is completely similar for the full inflammation pattern as the same elastin and collagen content was obtained after 90 days of growth and remodeling.

## References

[CR1] Ahmadzadeh H, Rausch MK, Humphrey JD (2018). Particle-based computational modelling of arterial disease. J R Soc Interface.

[CR2] Bellini C, Ferruzzi J, Roccabianca S, Di Martino ES, Humphrey JD (2014). A microstructurally motivated model of arterial wall mechanics with mechanobiological implications. Ann Biomed Eng.

[CR3] Berezowski M (2022). Early aortic growth in acute descending aortic dissection. Interact Cardiovasc Thorac Surg.

[CR4] Blount KJ, Hagspiel KD (2009). Aortic diameter true lumen, and false lumen growth rates in chronic type b aortic dissection. Am J Roentgenol.

[CR5] Braeu FA, Seitz A, Aydin RC, Cyron CJ (2017). Homogenized constrained mixture models for anisotropic volumetric growth and remodeling. Biomech Model Mechanobiol.

[CR6] Brandstaeter S, Fuchs SL, Biehler J, Aydin RC, Wall WA, Cyron CJ (2021). Global sensitivity analysis of a homogenized constrained mixture model of arterial growth and remodeling. J Elast.

[CR7] Brunet J (2023). In situ visualization of aortic dissection propagation in notched rabbit aorta using synchrotron X-ray tomography. Acta Biomater.

[CR8] Cyron CJ, Wilson JS, Humphrey JD (2014). Mechanobiological stability: a new paradigm to understand the enlargement of aneurysms?. J R Soc Interface.

[CR9] Cyron C, Aydin R, Humphrey J (2016) A homogenized constrained mixture (and mechanical analog) model for growth and remodeling of soft tissue Biomechanics and Modeling in Mechanobiology 15:1389–1403 10.1007/s10237-016-0770-9 10.1007/s10237-016-0770-9 PMC8378243 27008346

[CR10] Dake MD, Thompson M, van Sambeek M, Vermassen F, Morales JP, Investigators D (2013) DISSECT: A New Mnemonic-based Approach to the Categorization of Aortic Dissection Eur J Vasc Endovasc Surg 46:175–190 10.1016/j.ejvs.2013.04.029 10.1016/j.ejvs.2013.04.029 23721817

[CR11] Deplano V (2019). Mechanical characterisation of human ascending aorta dissection. J Biomech.

[CR12] Drews JD (2020). Spontaneous Reversal of Stenosis in Tissue-Engineered Vascular Grafts Sci Transl Med.

[CR13] Famaey N et al. (2018) Numerical simulation of arterial remodeling in pulmonary autografts ZAMM-Zeitschrift fur Angewandte Mathematik und Mechanik

[CR14] Fattori R et al. (2013) Survival After Endovascular Therapy in Patients With Type B Aortic Dissection A Report From the International Registry of Acute Aortic Dissection (IRAD) Jacc-Cardiovascular Interventions 6:876–882 10.1016/j.jcin.2013.05.003 10.1016/j.jcin.2013.05.003 23968705

[CR15] Gacek E, Mahutga RR, Barocas VH (2023). Hybrid discrete-continuum multiscale model of tissue growth and remodeling. Acta Biomater.

[CR16] Gheysen L, Maes L, Famaey N, Segers P (2023). Pulse wave velocity: A clinical measure to aid material parameter estimation in computational arterial biomechanics. J Biomech.

[CR17] He RM (2006). Characterization of the inflammatory and apoptotic cells in the aortas of patients with ascending thoracic aortic aneurysms and dissections. J Thorac Cardiovasc Surg.

[CR18] Holzapfel GA, Gasser TC, Ogden RW (2000). A new constitutive framework for arterial wall mechanics and a comparative study of material models. J Elast.

[CR19] Horgan CO, Murphy JG (2020). On the tension-compression switch hypothesis in arterial mechanics. J Mech Behav Biomed Mater.

[CR20] Horny L, Adamek T, Kulvajtova M (2014). Analysis of axial prestretch in the abdominal aorta with reference to post mortem interval and degree of atherosclerosis. J Mech Behav Biomed Mater.

[CR21] Horvat N, Virag L, Holzapfel GA, Soric J, Karsaj I (2019). A finite element implementation of a growth and remodeling model for soft biological tissues: Verification and application to abdominal aortic aneurysms. Comput Methods Appl Mech Eng.

[CR22] Humphrey JD (2013). Possible mechanical roles of glycosaminoglycans in thoracic aortic dissection and associations with dysregulated transforming growth factor-beta. J Vasc Res.

[CR23] Humphrey JD, Rajagopal KR (2002). A constrained mixture model for growth and remodeling of soft tissues. Math Models Methods Appl Sci.

[CR24] Iliopoulos DC, Kritharis EP, Giagini AT, Papadodima SA, Sokolis DP (2009). Ascending thoracic aortic aneurysms are associated with compositional remodeling and vessel stiffening but not weakening in age-matched subjects. J Thorac Cardiovasc Surg.

[CR25] Jubouri M (2022). Mid- and long-term outcomes of thoracic endovascular aortic repair in acute and subacute uncomplicated type B aortic dissection. J Card Surg.

[CR26] Karmonik C, Duran C, Shah DJ, Anaya-Ayala JE, Davies MG, Lumsden AB, Bismuth J (2012). Preliminary findings in quantification of changes in septal motion during follow-up of type B aortic dissections. J Vasc Surg.

[CR27] Kelly AM, Quint LE, Nan B, Zheng J, Cronin P, Deeb GM, Williams DM (2007). Aortic growth rates in chronic aortic dissection. Clin Radiol.

[CR28] Latorre M, Humphrey JD (2018). Modeling mechano-driven and immuno-mediated aortic maladaptation in hypertension. Biomech Model Mechanobiol.

[CR29] Latorre M, Bersi MR, Humphrey JD (2019). Computational modeling predicts immuno-mechanical mechanisms of maladaptive aortic remodeling in hypertension. Int J Eng Sci.

[CR30] Logghe G (2021). Outflow through Aortic Side Branches Drives False Lumen Patency in Type B Aortic Dissection Front Cardiovasc Med.

[CR31] Luo F, Zhou XL, Li JJ, Hui RT (2009). Inflammatory response is associated with aortic dissection. Ageing Res Rev.

[CR32] Maes L, Vervenne T, Van Hoof L, Jones EAV, Rega F, Famaey N (2023) Computational modeling reveals inflammation-driven dilatation of the pulmonary autograft in aortic position Biomechanics and Modeling in Mechanobiology:14 10.1007/s10237-023-01694-6 10.1007/s10237-023-01694-6 36764979

[CR33] Maes L, Famaey N (2023). How to implement constrained mixture growth and remodeling algorithms for soft biological tissues. J Mech Behav Biomed Mater.

[CR34] Miyahara S, Mukohara N, Fukuzumi M, Morimoto N, Murakami H, Nakagiri K, Yoshida M (2011). Long-term follow-up of acute type B aortic dissection: Ulcer-like projections in thrombosed false lumen play a role in late aortic events. J Thorac Cardiovasc Surg.

[CR35] Mousavi SJ, Farzaneh S, Avril S (2019). Patient-specific predictions of aneurysm growth and remodeling in the ascending thoracic aorta using the homogenized constrained mixture model. Biomech Model Mechanobiol.

[CR36] Nienaber CA, Kische S, Ince H, Fattori R (2011). Thoracic endovascular aneurysm repair for complicated type B aortic dissection. J Vasc Surg.

[CR37] Osada H, Kyogoku M, Ishidou M, Morishima M, Nakajima H (2013). Aortic dissection in the outer third of the media: what is the role of the vasa vasorum in the triggering process?. Eur J Cardio-Thorac Surg.

[CR38] Peterss S (2016). Changing Pathology of the Thoracic Aorta From Acute to Chronic Dissection Literature Review and Insights. J Am Coll Cardiol.

[CR39] Ramachandra AB, Latorre M, Szafron JM, Marsden AL, Humphrey JD (2020). Vascular adaptation in the presence of external support - A modeling study. J Mech Behav Biomed Mater.

[CR40] Sariola H, Viljanen T, Luosto R (1986). HISTOLOGICAL PATTERN AND CHANGES IN EXTRACELLULAR-MATRIX IN AORTIC DISSECTIONS. J Clin Pathol.

[CR41] Schlatmann TJM, Becker AE (1977). PATHOGENESIS OF DISSECTING ANEURYSM OF AORTA - COMPARATIVE HISTOPATHOLOGIC STUDY OF SIGNIFICANCE OF MEDIAL CHANGES. Am J Cardiol.

[CR42] Song JM (2007). Long-term predictors of descending aorta Aneurysmal change in patients with aortic dissection. J Am Coll Cardiol.

[CR43] Sueyoshi E, Sakamoto I, Uetani M (2009). Growth Rate of Affected Aorta in Patients With Type B Partially Closed Aortic Dissection. Ann Thorac Surg.

[CR44] Tolenaar JL (2013). Morphologic predictors of aortic dilatation in type B aortic dissection. J Vasc Surg.

[CR45] Trimarchi S (2013). Importance of false lumen thrombosis in type B aortic dissection prognosis. J Thorac Cardiovasc Surg.

[CR46] Valentin A, Humphrey JD (2009). Parameter Sensitivity Study of a Constrained Mixture Model of Arterial Growth and Remodeling J Biomech Eng-Trans ASME.

[CR47] Wang XW (2006). Increased collagen deposition and elevated expression of connective tissue growth factor in human thoracic aortic dissection. Circulation.

[CR48] Wang XH, Zhang HP, Cao L, He Y, Ma AR, Guo W (2020). The Role of Macrophages in Aortic Dissection. Front Physiol.

[CR49] Weisbecker H, Pierce DM, Regitnig P, Holzapfel GA (2012). Layer-specific damage experiments and modeling of human thoracic and abdominal aortas with non-atherosclerotic intimal thickening. J Mech Behav Biomed Mater.

[CR50] Xu LR, Burke A (2013). Acute Medial Dissection of the Ascending Aorta Evolution of Reactive Histologic Changes. Am J Surg Pathol.

[CR51] Yamauchi T, Masai T, Takano H, Shirakawa Y, Toda K, Sawa Y (2018). Osaka Cardiovasc Surg Res Grp OSC. Equations for Estimating the Predissected Diameter of the Descending Aorta from Computed Tomographic Images at the Onset of Aortic Dissection Journal of the American Heart Association.

